# Mechanisms underlying the increased susceptibility to initiation of cortical spreading depression in a genetic mouse model of migraine

**DOI:** 10.1186/s10194-025-02139-4

**Published:** 2025-11-13

**Authors:** Marina Vitale, Angelita Tottene, Maral Zarin Zadeh, Daniela Pietrobon

**Affiliations:** 1 Department of Biomedical Sciences, University of Padova, Padova, 35131 Italy; 2 Padova Neuroscience Center (PNC), University of Padova, Padova, 35131 Italy

**Keywords:** Migraine, Familial hemiplegic migraine, Cortical spreading depression, Spreading depolarization, Glutamate NMDA receptors, Ca_V_2.1 voltage-gated calcium channels

## Abstract

**Background:**

There is evidence from human and animal studies that cortical spreading depression (CSD) is the neurophysiological correlate of migraine aura and a trigger of migraine pain mechanisms. The mechanisms of CSD initiation in the brain of migraineurs remain unknown. Insights into this question can be obtained by studying the mechanisms underlying the facilitation of CSD initiation in genetic mouse models of migraine. Here, we investigated these mechanisms in knock-in mice carrying a mutation in the Ca_V_2.1 calcium channel, which causes pure familial hemiplegic migraine type 1 (FHM1 mice).

**Methods:**

Brief high-KCl puffs of increasing duration up to the threshold duration eliciting a CSD were applied on layer 2/3 whilst the membrane potential of a pyramidal neuron located very close to the site of KCl application and the intrinsic optic signal were simultaneously recorded in cortical slices from FHM1 mice. This was done before and after application of MK-801. After blocking the glutamate NMDA receptors (NMDARs), stimuli up to 21 times the control CSD threshold were applied.

**Results:**

A delayed activation of NMDARs above a critical threshold level is necessary for CSD initiation in FHM1 mice. This threshold level of NMDAR activation is quantitatively similar in FHM1 and WT mice, but is reached with a stimulus of much lower intensity and more rapidly in FHM1 mice, thus accounting for the facilitation of CSD initiation in these migraine mouse models. While, no matter the intensity of stimulation, Ca_V_ channels are necessary for CSD initiation, the necessity of NMDARs can be overcome by largely suprathreshold stimuli in FHM1 mice; however, these NMDAR-independent CSDs propagate much more slowly than the control CSDs, due to both a longer time needed to reach the threshold for CSD initiation after the beginning of the prodromal neuronal depolarization and a slower regenerative CSD depolarization.

**Conclusions:**

FHM1 mice are more susceptible to CSD initiation than WT mice because the critical threshold level of NMDAR activation, necessary for CSD initiation in both genotypes, is attained with stimuli of much lower intensity and more rapidly in FHM1 mice. Our findings give insights into potential mechanisms of CSD initiation in migraine.

## Background

Migraine is a common episodic neurological disorder with complex pathophysiology, that is characterized by recurrent attacks of typically throbbing and unilateral, often severe, headache with certain associated features, and by a global dysfunction in multisensory information processing [1–3]. In a third of patients, the headache is preceded by transient sensory disturbances, which are most frequently visual but may involve other modalities, the so-called migraine aura. There is evidence from human and animal studies that cortical spreading depression (CSD) is the neurophysiological correlate of migraine aura and also a trigger of the migraine pain mechanisms ( [2, 4, 5] and references therein) [6–10]. CSD is a slowly propagating self-sustaining wave of nearly complete depolarization of a sizable population of brain cells that lasts about one minute and silences brain electrical activity for several minutes (hence the name spreading depression) [4, 11–13]. A key unanswered question in migraine neurobiology concerns the mechanisms that make the brain of migraineurs susceptible to CSD.

Important insights into this question can be obtained by studying experimentally-induced CSD and the mechanisms underlying the facilitation of CSD initiation in genetic mouse models of migraine. Several knock-in mouse models for familial hemiplegic migraine (FHM), a rare monogenic form of migraine with aura, were generated [14, 15]. FHM is caused by either gain-of-function mutations in neuronal voltage-gated ion channels (the Ca^2+^ channel Ca_V_2.1 in FHM1 [16, 17] and the Na^+^ channel Na_V_1.1 in FHM3 [18, 19]) or by loss-of-function mutations in the predominantly astrocytic α_2_ Na^+^, K^+^ ATPase (α_2_NKA, in FHM2 [20, 21]). Some of these genetic mouse models carry mutations causing pure FHM, i.e. typical FHM attacks which, apart from the motor weakness during aura and the possible longer duration of the aura, resemble the attacks of common migraine with aura (MA); both FHM and MA attacks may alternate in patients and co-occur within families with pure FHM [22, 23]. Moreover, a transgenic mouse model carrying a mutation (in casein kinase 1δ) which causes MA associated with familial advanced sleep syndrome (FASPS) is also available [15, 24]. Despite mechanistically diverse mutations, all these genetic mouse models show an increased susceptibility to CSD induced by focal stimulation in vivo [24–34] and in vitro [32, 35–38], thus allowing an opportunity to investigate the underlying mechanisms.

In experimental models, CSD initiation in normally metabolizing brain tissue requires intense stimuli, which depolarize a minimum critical volume of tissue, increase the extracellular concentration of potassium ions [K^+^]_e_, and release glutamate (and other neurotransmitters) [4, 12]. Both computational models and experiments, measuring [K^+^]_e_ or glutamate at the CSD initiation site, support the idea that increases of [K^+^]_e_ and/or extracellular glutamate above critical values are key events for CSD ignition [4, 12, 31, 39–43]. The investigations of the mechanisms of facilitation of CSD initiation in genetic models of migraine carrying mutations causing pure FHM (FHM mice) or MA/FASPS (MA mice) support the role of excessive glutamatergic transmission as the likely ‘switch’ that mediates the all-or-none transition of the network to CSD [15]. Indeed, CSD facilitation rescue experiments showed that there is a causative link between increased Ca_V_2.1-dependent glutamatergic transmission at cortical synapses and facilitation of initiation and propagation of experimental CSD in FHM1 knock-in mice [35]. The MA mice showed enhanced glutamate release during high frequency stimulation consequent to reduced short-term depression at cortical synapses compared to wild-type (WT) mice, and the facilitation of CSD initiation in MA mice was rescued by eliminating the difference in short-term depression between the genotypes [38]. On the other hand, most of the facilitation of CSD initiation in FHM2 knock-in mice can be accounted for by their reduced rate of glutamate clearance at cortical excitatory synapses [31, 36]. Thus, overall the findings in the FHM1, FHM2, and MA mouse models support the conclusion that an excessive cortical glutamatergic transmission, due either to increased glutamate release or impaired glutamate clearance, underlies the facilitation of CSD initiation in these genetic mouse models of migraine.

Imaging of extracellular glutamate at the site of CSD initiation in awake head-fixed mice revealed that an increase in basal glutamate and in frequency of glutamate “plumes” precedes and may predict CSD initiation in both FHM2 and WT mice [31]. These findings support the idea of a threshold level of glutamate necessary for CSD ignition regardless of genotype. Due to their reduced rate of glutamate clearance, this threshold level is reached with a stimulus of lower intensity and more rapidly in FHM2 mice, thus accounting for the facilitation of CSD initiation [31]. We have recently shown that a critical threshold level of glutamate NMDA receptor (NMDAR) activation is necessary for CSD initiation by a brief focal high-KCl threshold stimulation in WT mice [44]. This threshold level is reached with a delay of few seconds, which coincides with the delay of CSD initiation relative to the rapid strong neuronal depolarization produced by the brief KCl puff [44]. These findings suggest that the enhanced activation of NMDARs is the critical mechanism downstream of the glutamate elevation which is necessary for CSD initiation. They also suggest the hypothesis that the same threshold level of NMDAR activation is required for CSD initiation in the genetic mouse models of migraine and WT mice, but this level is reached with stimuli of lower intensity in the migraine models, thus accounting for their increased susceptibility to CSD initiation.

Here, we tested this hypothesis in FHM1 mice, which carry the (pure FHM causing) R192Q mutation in the Ca_V_2.1 channel [27]. We show that indeed the threshold level of delayed NMDAR activation (which is critical for making the depolarization regenerative and initiate CSD at threshold stimulation) is similar in FHM1 and WT mice. However, in FHM1 mice, this critical level of NMDAR activation is reached with depolarizing stimuli that are largely subthreshold for CSD initiation in WT mice, thus accounting for the facilitation of CSD initiation in the migraine mouse model. We also show that, while voltage-gated Ca^2+^ channels (Ca_V_ channels) appear necessary for initiation of CSD (no matter the intensity of stimulation), the necessity of NMDARs for CSD initiation can be overcome by largely suprathreshold stimuli in FHM1 mice; however, these NMDAR-independent CSDs propagate much more slowly than the control CSDs, due to both a longer time needed to reach the threshold for CSD initiation after the beginning of the prodromal neuronal depolarization and a slower regenerative CSD depolarization.

## Methods

### Animals

Experiments were performed using WT C57BL6J and homozygous knock-in mice carrying the R192Q FHM1 mutation (RQ mice) with the same genetic background [27] or mice obtained by crossbreeding the WT and the homozygous RQ mice with homozygous FVB-Tg(GadGFP)45704Swn/J (GIN) mice expressing GFP in a subset of somatostatin-expressing interneurons (WT-GIN and RQ-GIN mice, 75% C57BL6J genetic background) [44–46]. Animals were housed in specific pathogen free conditions, maintained on a 12-h light/dark cycle, with free access to food and water. All experimental procedures involving animals and their care were carried out in accordance with Italian laws and policies (D.L. n. 26, March 14, 2014) and with the guidelines established by the European Community Council Directive (2010/63/UE) and were approved by the local authority veterinary services in Padova (Italy Aut. Min. 652/2015-PR and 340/2022-PR).

### Acute brain slice preparation

Acute coronal slices containing the somatosensory barrel cortex were prepared from postnatal day P17-20 male and female mice, as described in [36]. Briefly, animals were anesthetized with isoflurane and decapitated. The brain was quickly removed and put in an ice-cold cutting solution (in mM: 130 K gluconate, 15 KCl, 0.2 EGTA, 20 HEPES, 25 glucose, 2 kynurenic acid, 5 × 10^− 5^ minocycline, pH 7.4 with NaOH, oxygenated with 100% O_2_) [47]. 350 μm-thick slices were then cut on the coronal plane with a vibratome (VT1200S, Leica Biosystems, Germany) and were transferred for 1 min in a solution containing (in mM) 225 D-mannitol, 2.5 KCl, 1.25 NaH_2_PO_4_, 26 NaHCO_3_, 25 glucose, 0.8 CaCl_2_, 8 MgCl_2_, 2 kynurenic acid, 5 × 10^− 5^ minocycline, saturated with 95% O_2_ and 5% CO_2_. Slices were then maintained at 30 °C for 30 min in standard artificial cerebrospinal fluid saturated with 95% O_2_ and 5% CO_2_ (sACSF in mM: 125 NaCl, 2.5 KCl, 25 NaHCO_3_, 1.25 NaH_2_PO_4_, 1 MgCl_2_, 2 CaCl_2_, 25 glucose) plus 50 nM minocycline, and then transferred at room temperature in the same solution for a minimum of 30 min before being used for the experiment. All experiments were performed within 6 h from the mouse decapitation.

### Patch-clamp recordings

Whole-cell patch-clamp recordings were made following standard techniques. Electrical signals were recorded through a Multiclamp 700B amplifier and digitized using an Axon Digidata 1550 interface and pClamp software (Molecular Devices). Pipette resistance: 3–4 MΩ. Brain slices were continuously perfused in a submersion chamber with a fresh extracellular solution at room temperature at a flow rate of 3 ml/min using a peristaltic pump (Miniplus 3, Gilson). The extracellular solution contained: 125 mM NaCl, 3.5 mM KCl, 25 mM NaHCO_3_, 1.25 mM NaH_2_PO_4_, 0.5 mM MgCl_2_, 1 mM CaCl_2_, 25 mM glucose (saturated with 95% O_2_ and 5% CO_2_), as in [44]. This extracellular solution with relatively low Mg^2+^ (and Ca^2+^) concentrations was originally used [35] because it allows recording of spontaneous up and down states in slices similar (except for their longer duration and lower frequency) to those recorded in vivo in anesthetized animals (see in Fig. 7C two upstates in the control trace: one very close to the time of KCl application and one close to the beginning of the prodromal depolarization in this particular experiment). However, the CSD thresholds in WT and FHM1 mice and the facilitation of CSD induction in FHM1 mice measured in this medium and reported here (Fig. 2A: CSD threshold: 251 ± 37 ms (*n* = 16) in WT and 141 ± 19 ms (*n* = 13) in FHM1; 44% lower CSD threshold in FHM1 compared to WT) are similar to those measured in a medium containing 1 mM Mg^2+^ and 1.2 mM Ca^2+^ (as in [36]): CSD threshold: 253 ± 35 ms (*n* = 16) in WT and 149 ± 26 ms (*n* = 16) in FHM1; 41% lower CSD threshold in FHM1 compared to WT (our unpublished findings). Thus, most likely, the relatively low Mg^2+^ concentration in the extracellular medium does not affect the results and conclusions of our study.

**Fig. 1:**
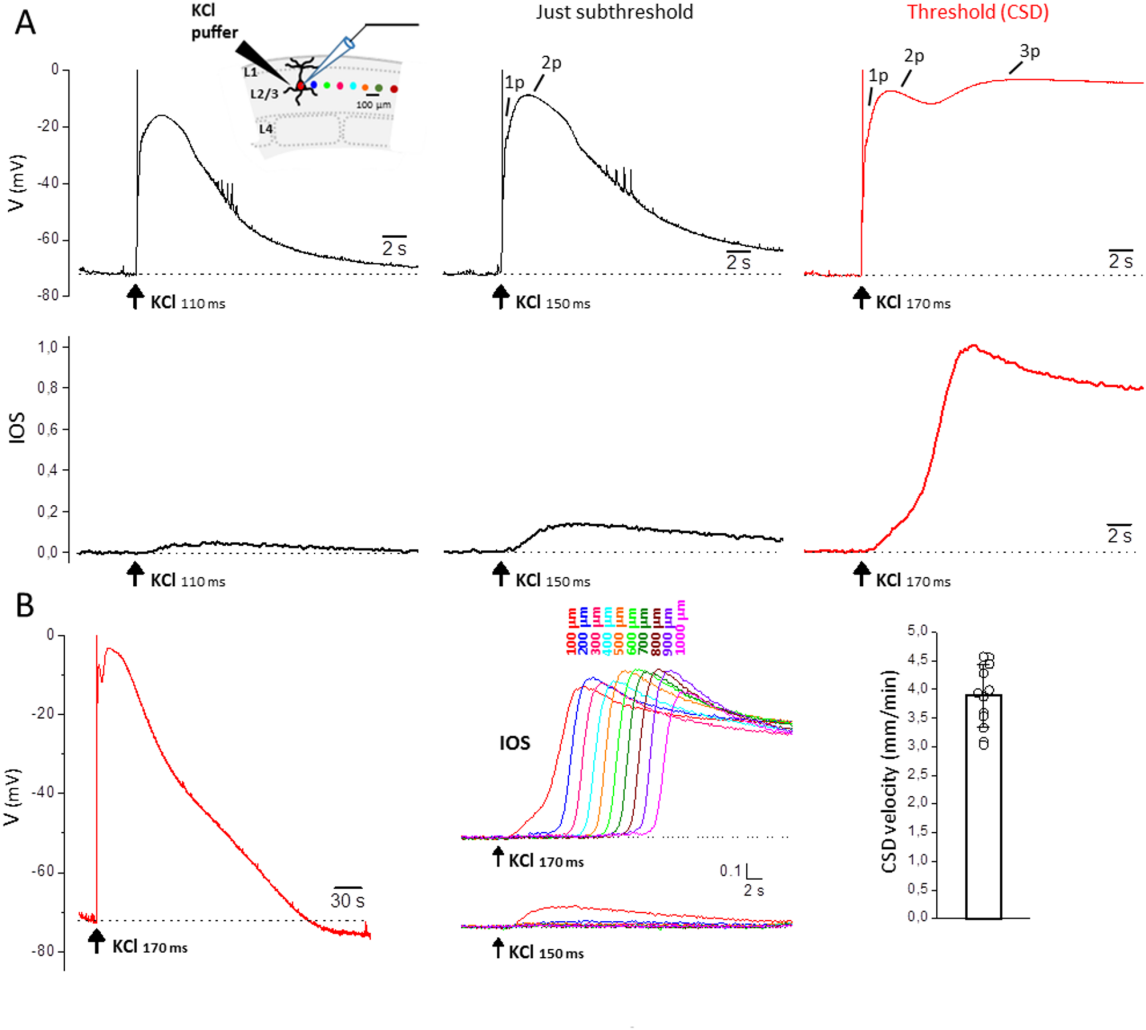
Neuronal voltage changes and IOS changes near the site of CSD induction elicited by CSD subthreshold and threshold KCl stimuli in FHM1 cortical slices. **A** High KCl puffs of increasing duration were applied onto the slice surface (L2/3) up to the threshold duration eliciting a CSD and, simultaneously, the IOS at the site of KCl application and at different distances from it (indicated by the colored dots in the inset) and the membrane potential of a L2/3 pyramidal neuron located at 100 μm from the KCl puffer were recorded (inset). Upper panels: representative membrane potential changes in a L2/3 pyramidal cell located near the KCl puffer (black traces) recorded in response to puffs of increasing duration (as indicated below the traces) up to the threshold duration eliciting a CSD (red trace). The voltage values of the first two peaks of the depolarization produced by the just subthreshold stimulus (indicated with 1p and 2p: -23 and − 9 mV, respectively) are similar to those of the first two peaks of the depolarization produced by the CSD threshold stimulus (-24 and − 7 mV, respectively), but the CSD threshold stimulus produces a third depolarization peak to -3 mV. Lower panels: normalized IOS changes recorded simultaneously at the same site in response to the same KCl puffs; the traces were normalized relative to the amplitude of the IOS change induced by the CSD threshold stimulus. The beginning of the steep IOS rise produced by the threshold KCl stimulus (red trace) occurs with a delay of 5.5 s from the KCl application and temporally coincides with the development of the third peak of the voltage change produced by the same stimulus. **B** Left: entire membrane potential change comprising the repolarization phase following the peak of the depolarization elicited by the CSD threshold KCl stimulus (same CSD trace as in panel A; HW = 98 s). Right: IOS changes recorded at different distances from the KCl puffer (as indicated above the traces and cf. inset in panel A for color code) in response to the CSD threshold (upper panel) and just subthreshold (lower panel) KCl stimulus in the same representative experiment. The steep IOS rise elicited by the CSD threshold stimulus propagates at a rate of 4.28 mm/min in this experiment. The bar plot shows the average rate of propagation of CSD, as obtained from the rate of propagation of the steep IOS rise elicited by CSD threshold stimuli

Membrane potential recordings were made from upper layer 2/3 (L2/3) pyramidal cells deeper than 45 μm from the slice surface. The cells were visualized using an upright microscope (Nikon Eclipse; Nikon, Tokyo, Japan) equipped with infrared light and infrared differential interference contrast optics (water-immersion objective 60×) and identified by their typical morphological pyramidal shape and the presence of a prominent apical dendrite and by their spiking pattern in response to 600 ms pulses of depolarizing current of increasing intensity [35]. The slices were used for recording only if more than 50% of the cells were alive at 45 μm depth in a 228 × 172 μm field. The internal solution contained: 114 mM K-gluconate, 6 mM KCl, 4 mM MgATP, 0.3 mM NaGTP, 10 mM Na-phosphocreatine, 10 mM HEPES, 30 mM sucrose (pH = 7.25 with KOH). The voltage values were measured against the reference electrode in the bath and were not corrected for the changes in extracellular voltage, which in our conditions were small: range − 3 to -5 mV (*n* = 6, *N* = 2) for both the main 2nd and 3rd peaks of the neuronal voltage changes measured at CSD threshold (cf. Fig. 1); these values should be subtracted to the measured voltages to obtain the correct membrane potential. The voltage values in the Figures and text were also not corrected for the measured liquid junction potential of -10 mV; this value should be added to all voltages to obtain the correct membrane potentials.

### Cortical spreading depression

CSD was elicited in acute cortical coronal slices as in [35]. Briefly, the brain slices were placed into a submersion chamber and continuously perfused with fresh extracellular solution at room temperature at a flow rate of 3 ml/min. Brief pressure-ejection pulses of 3 M KCl (0.5 bar) of increasing duration (at 5 min intervals) were applied through a glass micropipette (resistance ranging from 0.20 to 0.23 MΩ) onto the slice surface on L2/3, using a PDES-02DX pneumatic drug ejection system (Npi Electronic GmbH, Tamm, Germany), until a CSD was elicited. For each KCl stimulus of increasing duration, the intrinsic optic signal (IOS) changes at the tip of the KCl puffer and at increasing distances from it and the membrane potential of a L2/3 pyramidal cell located at 100 μm from the tip of the KCl puffer were simultaneously recorded. CSD was detected by the typical long-lasting depolarization to almost 0 mV in the patch-clamped L2/3 pyramidal cell and/or by the typical propagating steep change in IOS (Fig. 1). The duration of the first pulse eliciting a CSD was taken as CSD threshold and the rate of horizontal spread of the change in IOS as CSD velocity. IOS was recorded using a CMOS camera (Basler ace acA1920-155 μm USB 3.0, Basler, Germany) connected to the upright microscope (Nikon Eclipse; 10× magnification). Images were recorded at 200 ms intervals as 1920 × 1200 pixels images (pixel size: 1.27 μm). MBF ImageJ software was used for the offline analysis of the digitalized images. The IOS change is expressed as change in light transmittance relative to the background signal.

The effect of blocking the NMDARs on the depolarization and the IOS elicited by CSD subthreshold and threshold KCl stimuli was investigated by first measuring the control CSD threshold as described above, and then, after 30 min, measuring the depolarization and the IOS change induced by the same KCl stimuli in the presence of a saturating concentration of MK-801 (which had been perfused for 20–25 min). A CSD-inducing KCl stimulus was considered a threshold stimulus if the largest subthreshold KCl stimulus applied during the measurement of the control CSD had a duration only 20–40 ms lower than the threshold stimulus (i.e. it was just subthreshold). The effect of blocking the NMDARs (with 50 µM MK-801) or the Ca_V_ channels (with 5 mM Ni^2+^) on the depolarization and/or the IOS elicited by largely suprathreshold KCl stimuli was investigated by first measuring the control CSD threshold as described above, and then, after 30 min, measuring the depolarizations and/or the IOS changes induced by KCl stimuli of duration up to 21 times longer than the CSD threshold stimulus in the presence of the specific channel blocker (which had been perfused for 20–25 min). To study the properties of the CSD depolarizations elicited in FHM1 mice by largely suprathreshold stimuli after blocking NMDARs, we recorded the voltage changes of neurons located at ≥ 600 μm from the KCl puffer (and the IOS change at the same location) in response to CSD threshold KCl puffs in control and to both threshold and suprathreshold KCl stimuli after MK-801 perfusion.

We have previously reported that after 30 min from the induction of the CSD in control conditions the post-CSD refractory period has ended and there are no other relevant post-CSD effects in cortical slices from WT mice [44]. We verified that this holds also in cortical slices from FHM1 mice by performing some control IOS experiments. Indeed, as found in WT slices, the CSD threshold in FHM1 slices remained unaltered after 30 min of sham (extracellular solution) perfusion following the assessment of the control CSD threshold (135 ± 10 ms sham vs. 130 ± 23 ms control, *n* = 4, *N* = 4, Wilcoxon signed rank test, *p* = 0.8) and the CSD velocity was only slightly reduced as a trend (3.23 ± 0.27 mm/min sham vs. 3.82 ± 0.30 mm/min control, *n* = 4, *N* = 4, paired t-test, *p* = 0.07).

Series resistance was monitored throughout the experiment; experiments with series resistance > 30 MΩ were excluded from the data. The fact that often the series resistance increased and/or the seal deteriorated after the induction of the control CSD limited the numerosity of the experiments in which we could record the membrane potential of the pyramidal cell before and after drug perfusion in response to both subthreshold and threshold stimulation.

**Fig. 2:**
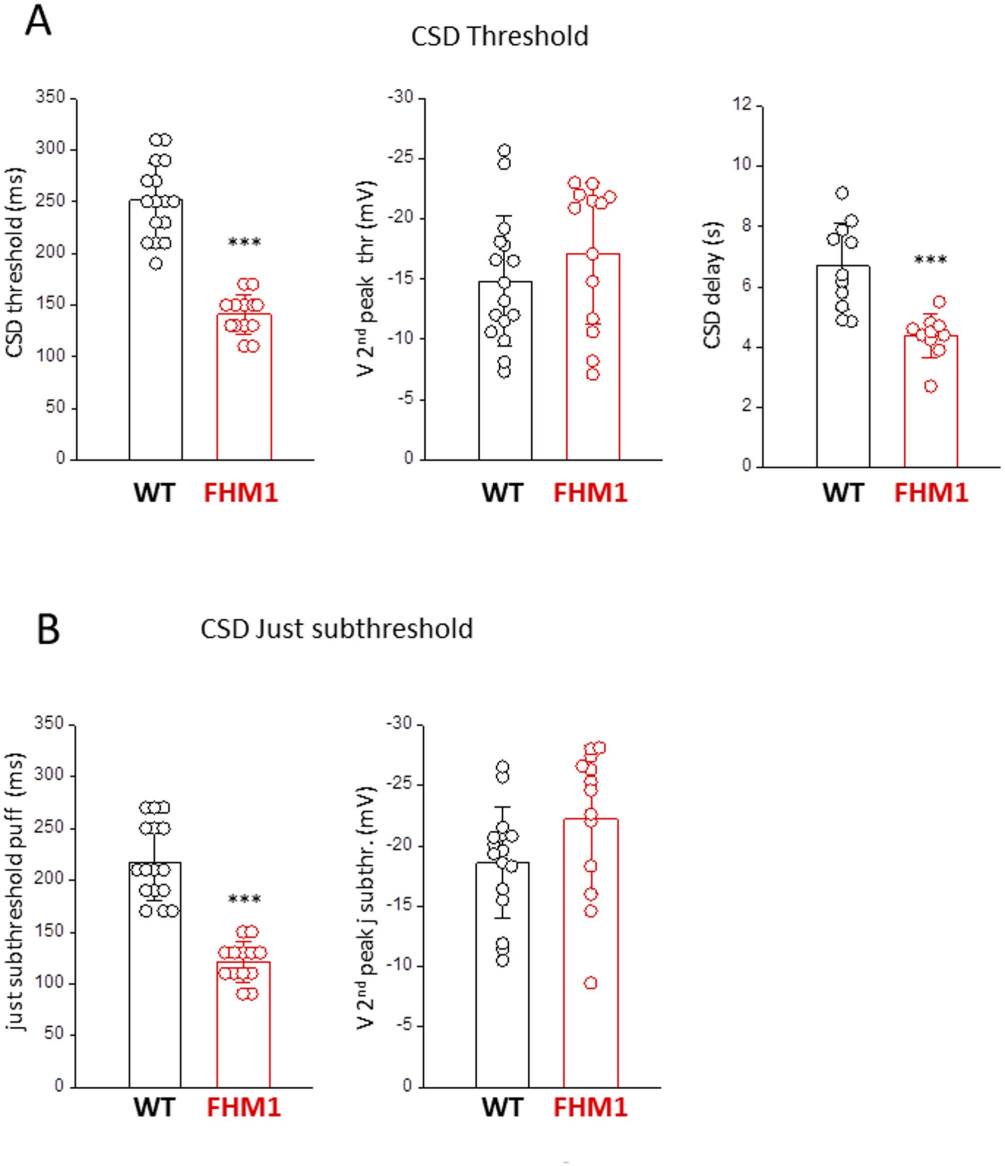
KCl stimuli of much lower intensity in FHM1 compared to WT mice produce similar neuronal depolarizations near the site of CSD induction in the two genotypes. **A** The duration of the first KCl pulse eliciting a CSD (CSD threshold) is lower in FHM1 compared to WT mice [44](left panel; MW test, *p* = 0.000005). Despite this, the amplitude of the 2nd peak of the early neuronal depolarization preceding CSD (cf. Fig. 1A) is similar in FHM1 and WT mice [44] (middle panel; t test, *p* = 0.3), and the beginning of CSD, as obtained from the beginning of the steep IOS rise, occurs with a shorter delay relative to the time of KCl application in FHM1 compared to WT mice [44] (right panel; t test, *p* = 0.0002). **B** Despite the smaller just subthreshold KCl stimulus in FHM1 mice (left panel; MW test, *p* = 0.000005), the amplitude of the 2nd peak of the just subthreshold depolarization (cf. Fig. 1A) was similar in FHM1 and WT mice [44] (right panel; MW test, *p* = 0.09)

**Fig. 3:**
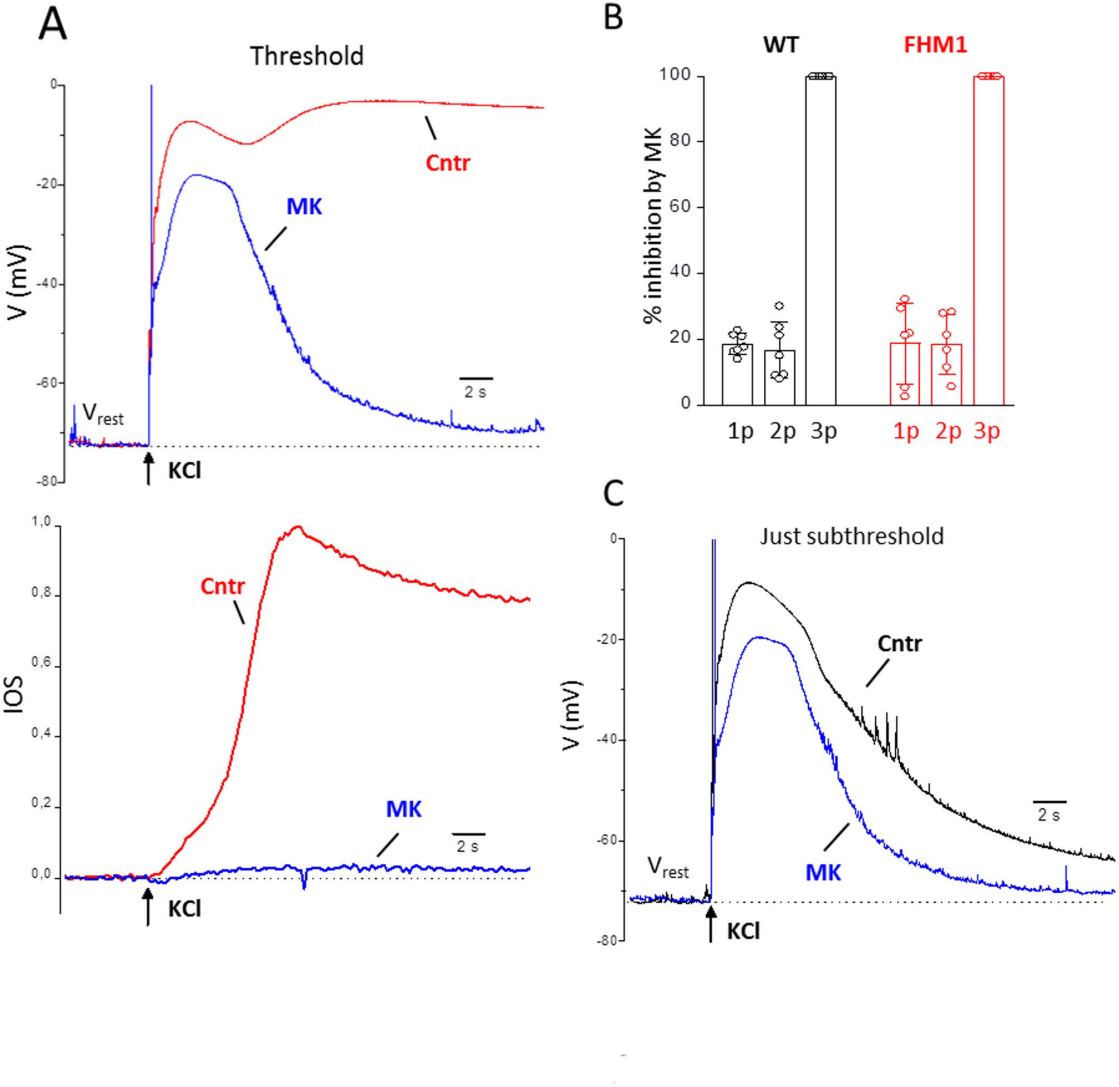
Effect of blocking the NMDARs on the neuronal depolarizations elicited near the site of CSD induction by CSD threshold and just subthreshold KCl stimuli in FHM1 mice. **A** Representative membrane potential traces recorded in response to a CSD threshold KCl stimulus in a L2/3 pyramidal cell located near the KCl puffer (as in Fig. 1) in the absence (ctrl, red trace) and presence (MK, blue trace) of MK-801 (50 µM) in a FHM1 slice (upper panel). The corresponding, simultaneously recorded, IOS changes are shown in the lower panel. MK inhibited only 32% and 17% of the amplitudes (relative to the resting potential) of the 1st and 2nd peak of the depolarization elicited by the CSD threshold stimulus, but completely eliminated the third CSD peak as well as the steep CSD IOS rise. **B** Average percentages of inhibition by MK-801 (50 µM) of the peak amplitudes (relative to resting potential) of the depolarizations elicited in L2/3 pyramidal cells located near the KCl puffer by CSD threshold KCl stimuli were similar in FHM1 and WT mice [44] (t test *p* = 1 and 0.7 for 1p and 2p, respectively). 1p, 2p and 3p refer to the 1st, 2nd and 3rd (CSD) peaks of the depolarization, as indicated in Fig. 1A. **C** Representative membrane potential traces recorded in response to a CSD just subthreshold KCl stimulus (20 ms shorter than the threshold stimulus) in the same L2/3 pyramidal cell of panel A in the absence (ctrl, black trace) and presence (MK, blue trace) of MK-801 (50 µM). MK inhibited 34% and 16% of the amplitudes of the 1st and 2nd peak of the depolarization elicited by the just subthreshold stimulus, and completely eliminated the shoulder after the 2nd peak, thus decreasing the duration (at half amplitude) of the just subthreshold depolarization from 9.8 to 7.5 s

**Fig. 4:**
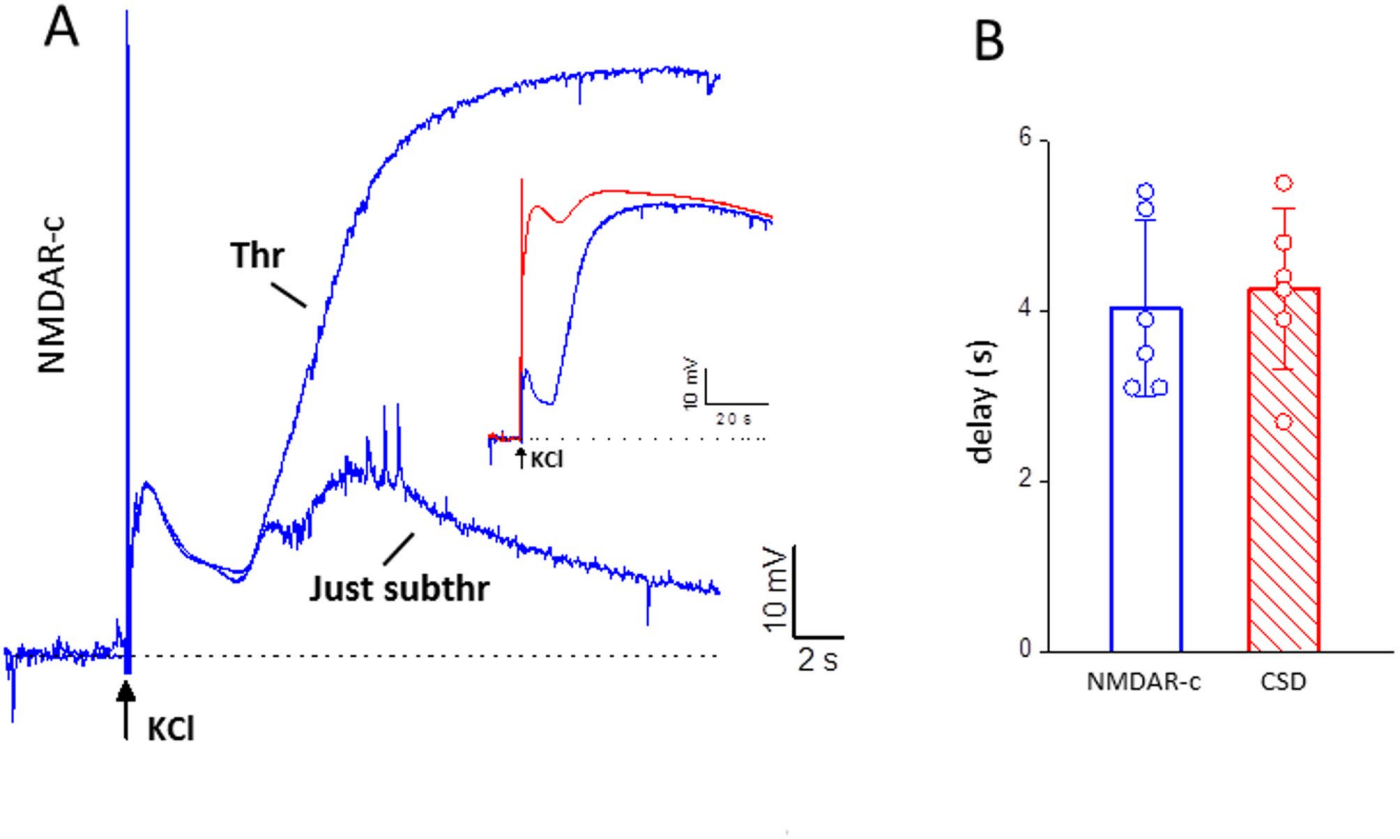
The delayed opening of a sufficient number of NMDARs underlies the initiation of CSD by a threshold stimulus in FHM1 mice. **A** NMDAR-dependent components (NMDAR-c) of the neuronal depolarizations elicited by the threshold (Thr) and just subthreshold (Just subthr) KCl stimuli, obtained by subtracting the voltages traces recorded after application of MK-801 to the control voltage traces in the absence of drug (same representative experiment as in Fig. 3). At CSD threshold stimulation, a large delayed NMDAR-c develops with a delay of 5.4 s from the application of KCl, which is similar to the delay of CSD ignition as derived from the steep IOS rise (5.5 s, cf. Fig. 3A); the inset shows that this component (blue trace) overlaps with and has the same duration of the CSD depolarization (red trace 3rd peak, cf. Fig. 1). At just subthreshold stimulation a delayed NMDAR-c of much smaller amplitude than at threshold (13 vs. 56 mV) develops with a similar delay (5.0 s). **B** The average delay (from the time of the KCl stimulus) of the late NMDAR-c of the depolarization elicited by threshold KCl stimuli is similar to the delay of CSD initiation (from the time of the KCl stimulus), derived from the beginning of the steep IOS rise in the same experiments (t test, *p* = 0.7)

**Fig. 5:**
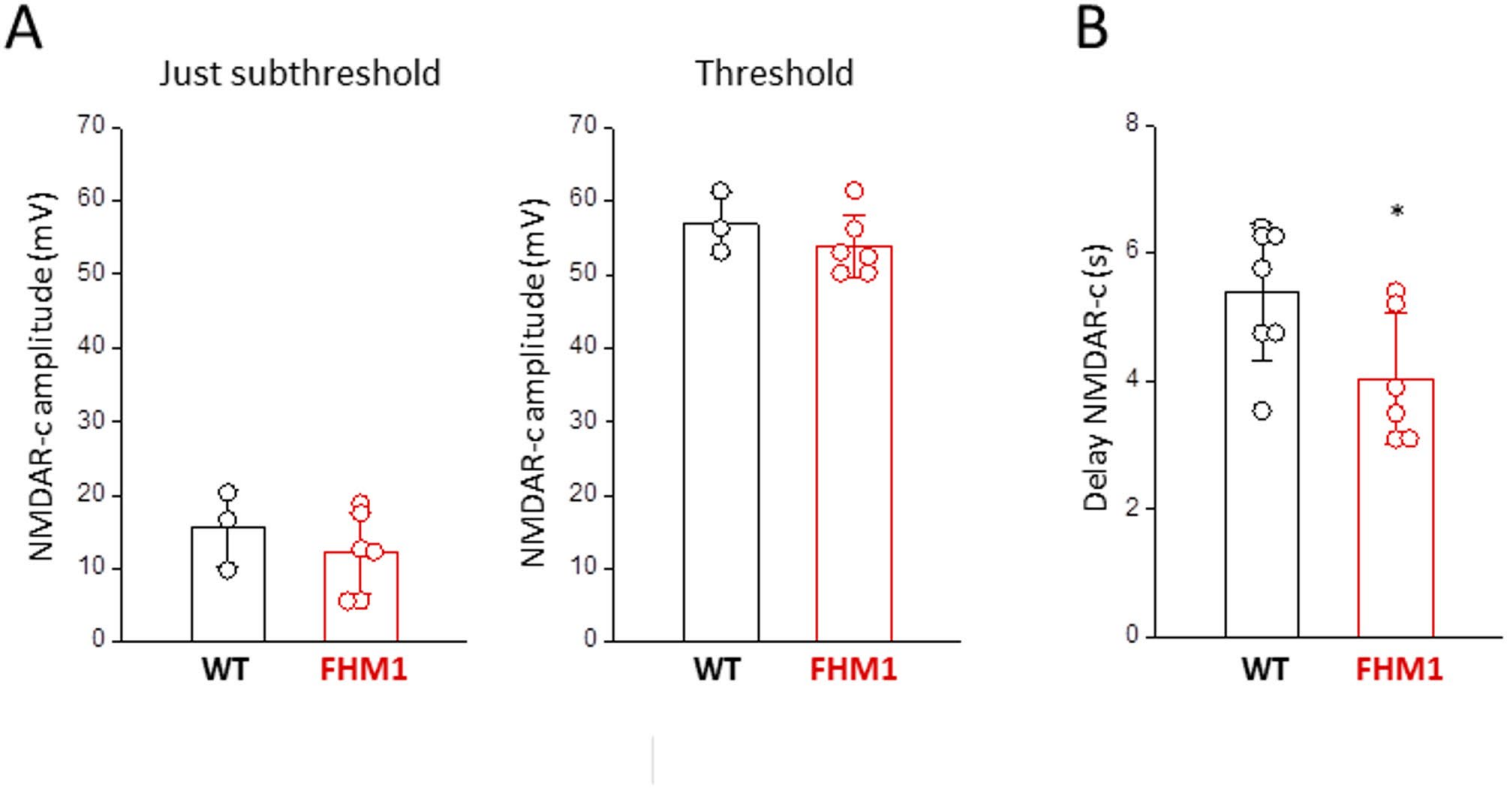
A similar threshold level of NMDAR activation underlies CSD initiation in FHM1 and WT mice, but this critical level is reached with a stimulus of lower intensity and more rapidly in FHM1 compared to WT mice. **A** The delayed NMDAR-dependent components (NMDAR-c) of the neuronal depolarization at just-subthreshold and threshold stimulations have both similar amplitudes in FHM1 and WT mice [44] (t-test: *p* = 0.4 and *p* = 0.47 for just-subthreshold and threshold, respectively), despite the lower stimulation intensities in FHM1 mice (cf. Fig. 2). **B** The delay of the late NMDAR-dependent component of the neuronal depolarization relative to the rapid depolarization produced by the threshold KCl stimulus is shorter in FHM1 compared to WT mice [44], despite the lower stimulation intensity (t test, *p* = 0.04)

**Fig. 6:**
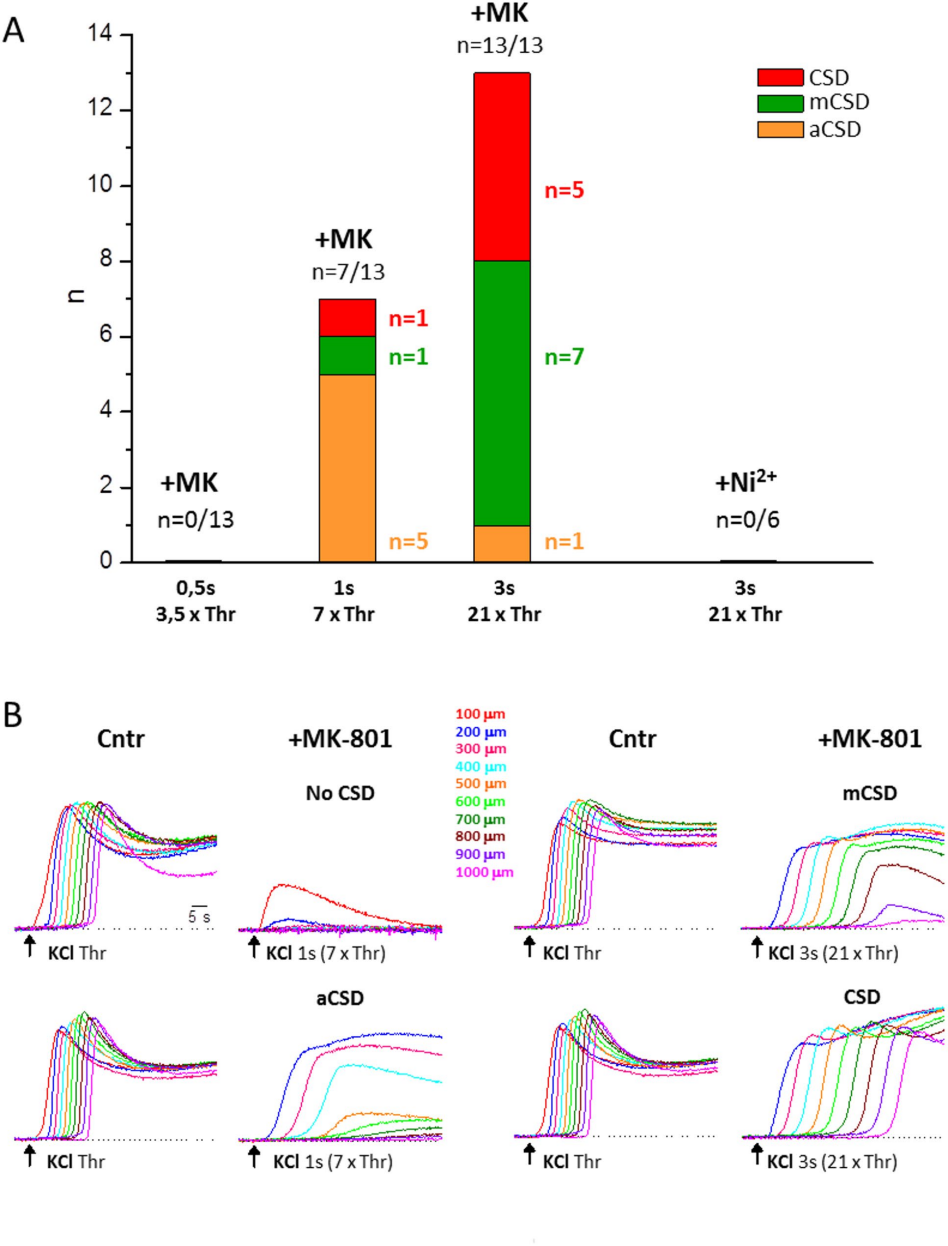
Besides activation of NMDARs, other Ca_V_2.1-dependent processes contribute to CSD initiation by very intense suprathreshold stimulation in FHM1 mice. **A** Number of CSDs (red), mCSDs (mini CSDs propagating to a distance < 1 mm but > 0.5 mm, green), a CSDs (aborted CSDs propagating < 0.5 mm, orange) elicited in the presence of MK (50 µM) by suprathreshold KCl puffs of duration 3.5, 7 and 21 times the control CSD threshold (*n* = 13; *N* = 11) or elicited in the presence of Ni^2+^ (5 mM) by suprathreshold KCl puffs of duration 21 times the control CSD threshold (*n* = 6; *N* = 3). **B** IOS changes recorded at different distances from the KCl puffer (color code as indicated) in response to the CSD threshold KCl stimulus in control and to the indicated suprathreshold KCl stimuli after perfusion of MK in four representative experiments in which, in the presence of MK, no CSDs (1st row traces on the left), an aCSD (2nd row traces on the left), a mCSD (1st row traces on the right) or a CSD propagating throughout the slice (2nd row traces on the right) were elicited. The mCSDs and CSDs elicited in the presence of MK propagated at a lower velocity than the control CSD: 1.32 mm/min vs. 3.59 mm/min and 1.24 mm/min vs. 3.93 mm/min for the representative mCSD and CSD shown in the right panel

**Fig. 7:**
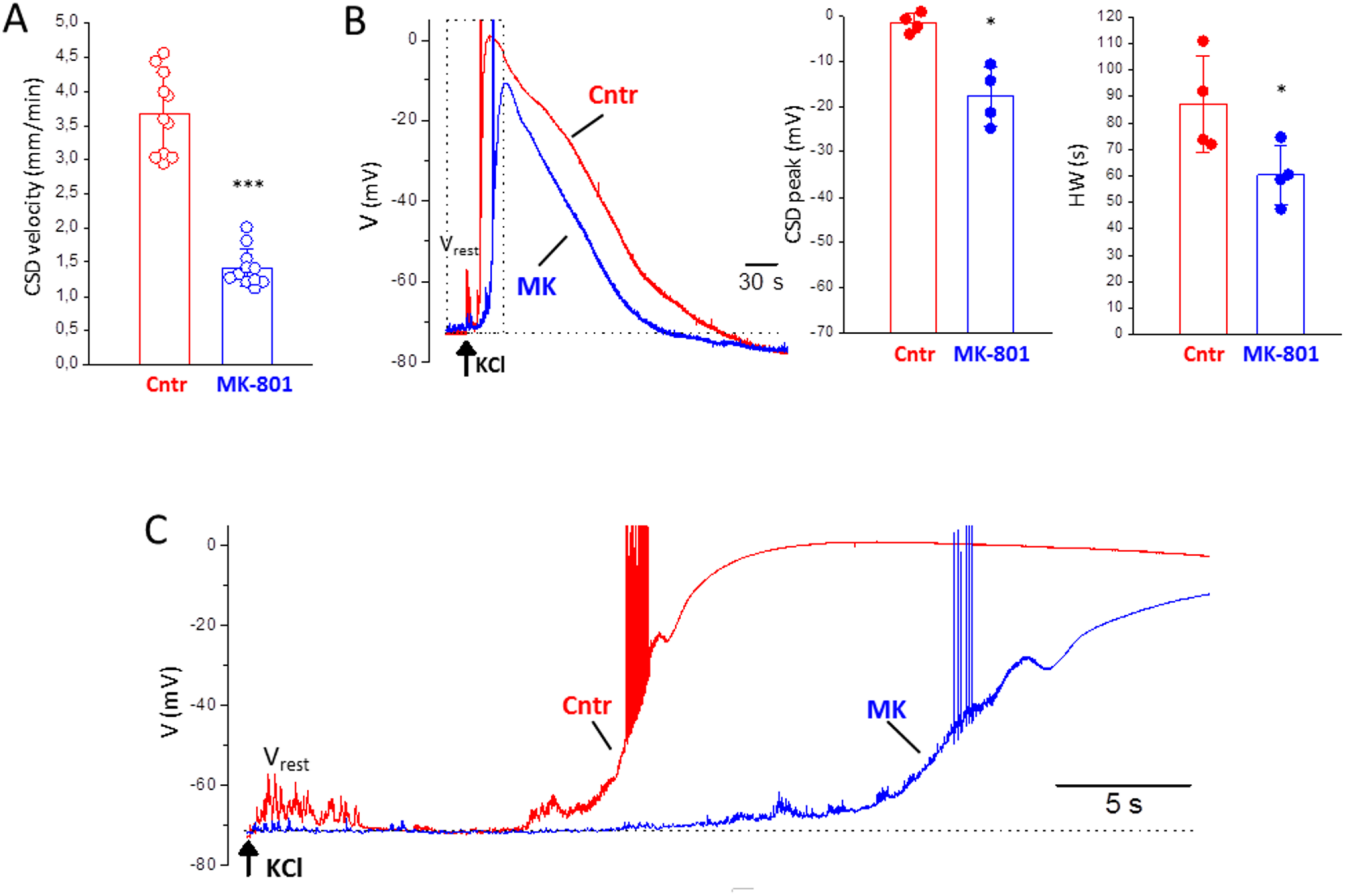
The CSD depolarizations elicited by very intense stimulation in FHM1 mice after block of NMDARs propagate at a lower velocity and are smaller, shorter and slower than the control CSDs. **A** The CSDs elicited by largely suprathreshold (relative to control) stimulation after block of NMDARs in FHM1 mice propagate at a much slower rate than the control CSDs elicited by threshold stimulation (paired t test, *p* = 0.000002). **B** Left panel: representative traces of the CSD depolarizations recorded in a neuron located at 665 μm from the KCl puffer in control with threshold stimulation (red trace) and after perfusion of MK-801 (MK 50 µM) with 6 times control CSD threshold stimulation (blue trace). Right panels: both the peak voltage and the duration (at half amplitude, HW) of the CSDs recorded after NMDAR block are lower than those of the control CSDs (paired t tests, *p* = 0.01 and *p* = 0.04, respectively). **C** A portion of the representative CSD traces (indicated by the dotted lines) in B is shown on an expanded time scale

**Fig. 8:**
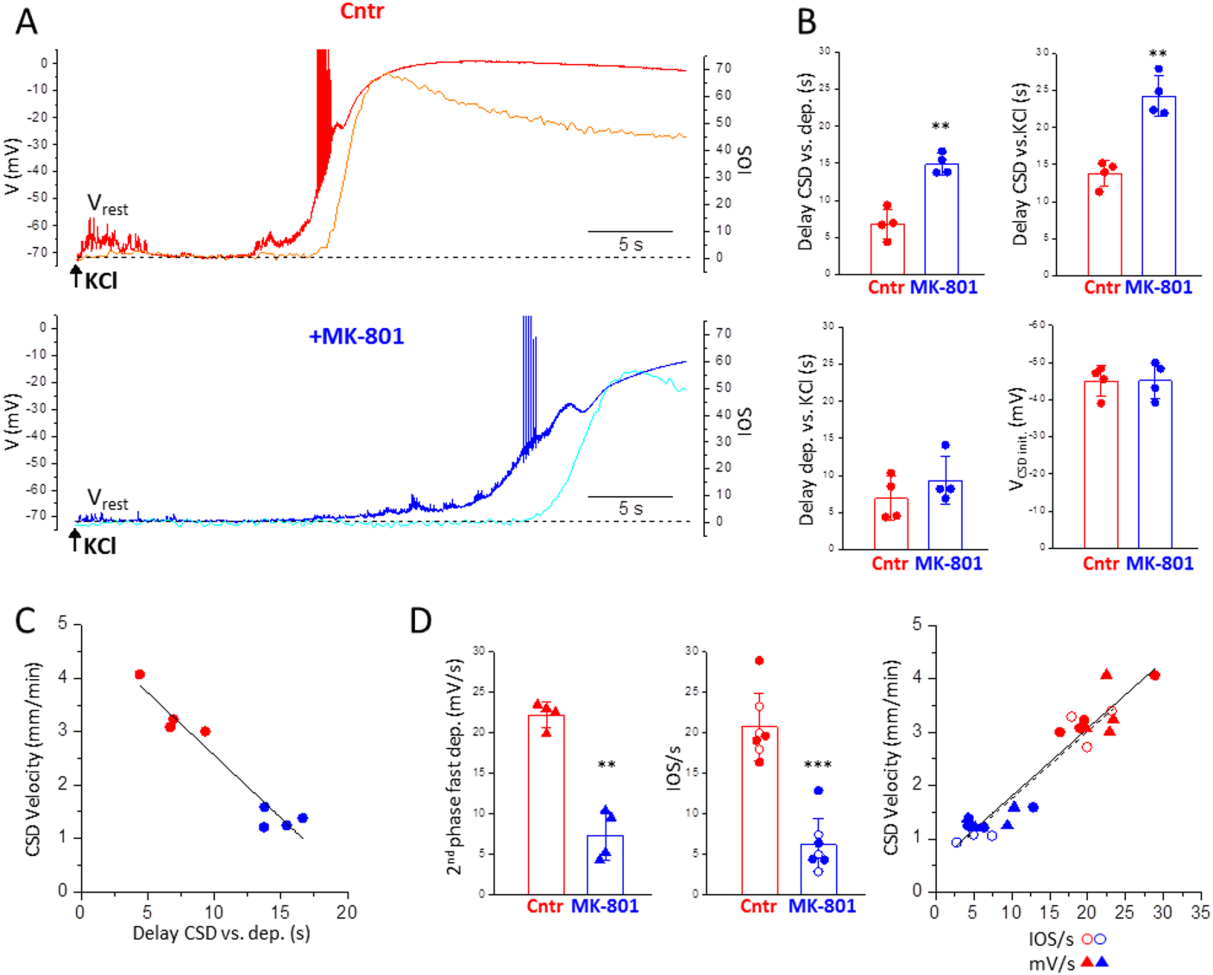
The slower velocity of CSD propagation after NMDAR block in FHM1 mice is due to both a longer delay of CSD initiation relative to the beginning of the prodromal neuronal depolarization and a slower regenerative CSD depolarization. **A**. Representative traces of the voltage and IOS changes recorded simultaneously at the location of the recorded neuron (at 665 μm from the KCl puffer) in control with CSD threshold stimulation (upper panel: red and orange traces for V and IOS, respectively) and after perfusion of MK-801 (50 µM) with 6 times control threshold stimulation (lower panel: blue and cyan traces for V and IOS, respectively). **B**. The delays with which the steep IOS rise initiates relative to the beginning of the prodromal depolarization (delay CSD vs. dep) and relative to the time of the high KCl puff (delay CSD vs. KCl) are both longer after block of NMDARs compared to control (upper panels: paired t tests, *p* = 0.003 and *p* = 0.002, respectively). In contrast, the delay of the beginning of the neuronal depolarization relative to the KCl puff (delay dep vs. KCl) is not significantly longer after NMDAR block compared to control (lower left panel: paired t test *p* = 0.09). The membrane potential at which CSD initiates (V_CSD init_) is identical in control and after NMDAR block (lower right panel: paired t test, *p* = 0.94). **C**. CSD velocity of propagation as a function of the delay of CSD initiation relative to the beginning of the prodromal depolarization in individual experiments in control (red) and after block of NMDARs (blue). The black line represents the best fit with a linear equation (slope: -0.235 ± 0.025). **D**. Left panels: both the second phase of the fast CSD depolarization and the propagating fast IOS change are slower in the presence of MK-801 than in control (paired t tests, *p* = 0.0015 and *p* = 0.00001, respectively). Right panel: velocity of CSD propagation as a function of the change per second of the fast propagating IOS (IOS/s: circles ●, ○) and as a function of the change per second of the fast regenerative depolarization (2nd phase mV/s: triangles ▲) in individual experiments in control (red) and in the presence of MK-801 (blue). The filled symbols refer to the same experiments as in the other panels of Fig. 8 while the empty circles refer to additional experiments for which only IOS recordings were available. The black continuous and dashed lines represent the best fits with linear equations, which give identical slopes for CSD rate vs. IOS/s (slope 0.13 ± 0.01) and CSD rate vs. depolarization/s (2nd phase; slope 0.13 ± 0.02)

The CSD threshold and velocity measured in RQ and RQ-GIN mice were similar (CSD threshold: 160 ± 39 ms, *n* = 8, *N* = 6 vs. 143 ± 18 ms, *n* = 12, *N* = 12, Mann-Whitney test *p* = 0.28; CSD velocity: 3.40 ± 0.57 mm/min, *n* = 8, *N* = 6 vs. 3.85 ± 0.55 mm/min, *n* = 12, *N* = 12, t-test *p* = 0.09). Hence, in the Results we refer to them as FHM1 mice without distinction, although most of the experiments in Figs. 1, 2, 3, 4, 5 and 6 were on RQ-GIN mice, while those on Figs. 7 and 8 were on RQ mice.

### Statistics

Statistical analyses were performed with Statgraphics centurion XVII software (RRID: SCR_015248). After assessing for normal distribution (using the Shapiro–Wilk normality test), comparison between two groups was made using two-tailed unpaired or paired *t* test for normally distributed data and the Mann–Whitney (MW) or Wilcoxon signed rank tests for nonparametric data. Equal variances were assumed. Data are given in the text and figures as mean ± SD. The significance level was set at *p* < 0.05 (*P < 0.05; **P < 0.01; ***P < 0.001).

The number *n* of observations (reported in the text and legends to figures) indicates the number of cells or slices recorded from, and the number *N* indicates the number of mice from which the data were obtained. No statistical methods were used to choose sample sizes that were estimated based on previous experience and are in line with those in the literature.

## Results

To test the hypothesis that the same threshold level of NMDAR activation is required for CSD initiation in FHM1 and WT mice, but this level is reached with stimuli of lower intensity in FHM1 mice, we performed in acute cortical slices from WT [44] and FHM1 mice the following experiment. We applied high KCl puffs of increasing duration (i.e. depolarizing stimuli of increasing intensity) onto the slice surface (L2/3) up to the threshold duration eliciting a CSD, and simultaneously recorded the membrane potential of a pyramidal neuron located very close (100 μm) to the site of KCl application and the IOS at this site and at different distances from it (Fig. 1A inset, colored dots). This was done before and after application of MK-801 (50 µM) to block NMDARs.

The CSD subthreshold KCl stimuli produce neuronal depolarizations that increase and change shape with increasing puff duration (Fig. 1A, black traces in upper panel). The depolarization produced by the just subthreshold stimulus (20 ms smaller puff duration than the CSD threshold) is characterized by two depolarization peaks (indicated with 1p and 2p in Fig. 1A) and a hint of a third peak which becomes evident as a shoulder following the first two peaks and/or as a prolongation of the overall depolarization. The CSD depolarization induced by the threshold KCl stimulus (Fig. 1A, B red traces) is characterized by a third peak to almost 0 mV (-3.6 ± 2.5 mV, *n* = 13, *N* = 13) following the first two peaks, and by a much longer duration (87 ± 15 s duration at half amplitude, HW) than the just subthreshold depolarization (7.8 ± 1.7 s HW duration; paired t-test: *p* = 1 × 10^− 9^). The small, slow IOS change elicited by the just subthreshold stimulus (Fig. 1A, lower panel) rapidly declines with increasing distance from the KCl puffer (Fig. 1B). In contrast, the CSD threshold KCl stimulus induces the steep large IOS change typical of CSD (Fig. 1A, red trace in the lower panel), whose amplitude does not decrease with distance (Fig. 1B) and propagates at an average rate of 3.89 ± 0.55 mm/min (Fig. 1C). The steep IOS rise typical of CSD (measured at the location of the recorded pyramidal cell) begins with a certain delay after the KCl application and is preceded by a non-propagating slower, smaller IOS rise, similar to that produced by the just subthreshold depolarization (Fig. 1A, B). The beginning of CSD, as obtained from the beginning of the steep IOS rise, occurs with a delay of 4.4 ± 0.7 s relative to the time of KCl application (*n* = 10; *N* = 10; Fig. 2A, right panel). Comparison with the simultaneously recorded neuronal membrane potential shows that the beginning of CSD does not coincide with the nearly immediate neuronal depolarization produced by the CSD threshold KCl puff. It occurs after the first two depolarization peaks and appears to temporally overlap with the development of the third depolarization peak (Fig. 1A).

The changes in amplitude and shape of the depolarizations produced by increasing subthreshold stimulations as well as the properties of the CSD depolarization (and threshold IOS change) in comparison with those of the just subthreshold depolarization (and just subthreshold IOS change) in FHM1 mice (Fig. 1A upper panel) are quite similar to those that we described in WT mice [44]. However, in agreement with the original findings of [35], the CSD threshold, as measured from the duration of the first pulse eliciting a CSD, was lower in FHM1 compared to WT mice: 141 ± 19 ms (*n* = 13, *N* = 13) in FHM1 vs. 251 ± 37 ms (*n* = 16, *N* = 15) in WT [44] (Fig. 2A, left panel). Despite the much smaller threshold KCl stimulus in FHM1 mice, the amplitude of the 2nd peak of the early depolarization preceding CSD was similar in FHM1 and WT mice (membrane potential of the 2nd peak: -17 ± 6 mV in FHM1 and − 15 ± 5 mV in WT [44]) (Fig. 2A, middle panel). Similarly, despite the much smaller just subthreshold KCl stimulus in FHM1 mice (121 ± 19 ms in FHM1 vs. 218 ± 37 ms in WT [44]), the amplitude of the 2nd peak of the just subthreshold depolarization was similar in FHM1 and WT mice (membrane potential of the 2nd peak: -22 ± 6 mV in FHM1 and − 19 ± 5 mV in WT [44]) (Fig. 2B). Thus, similar neuronal depolarizations in FHM1 and WT mice are produced by KCl stimuli of much lower intensity in FHM1 compared to WT mice. Moreover, interestingly, despite the smaller threshold KCl stimulus in FHM1 compared to WT mice, the beginning of CSD, as obtained from the beginning of the steep IOS rise, occurs with a shorter delay relative to the time of KCl application in FHM1 compared to WT mice: 4.4 ± 0.7 s (*n* = 10, *N* = 10) in FHM1 vs. 6.7 ± 1.4 s (*n* = 11, *N* = 11) in WT (Fig. 2A, right panel).

The effect of the NMDAR antagonist MK-801 (MK) on the neuronal depolarizations elicited by CSD threshold and subthreshold KCl stimuli at the site of CSD induction in FHM1 mice was studied by measuring them in control and after perfusion of the drug for 20–30 min, as in [44] (see Methods). As found in WT mice [44], blocking the glutamate NMDARs in FHM1 mice (i) prevented CSD initiation at threshold stimulation, as shown by the complete elimination of the third depolarization peak and the steep IOS rise, and (ii) produced a relatively small inhibition of the early depolarization preceding CSD initiation (while it almost completely inhibited the slow IOS rise preceding CSD initiation) (*n* = 6, *N* = 6, Fig. 3A, B). The complete inhibition of CSD initiation by threshold KCl stimuli after perfusion with MK was observed in additional 7 experiments (*N* = 6) with only IOS recordings. Despite the lower intensity of threshold stimulation in FHM1 mice (Fig. 2A), the inhibition of the early depolarization preceding CSD was similar in FHM1 and WT mice: 19 ± 12% and 19 ± 9% inhibition of the 1st and 2nd peak amplitudes relative to the resting potential, respectively, in FHM1 mice (*n* = 6; *N* = 6) vs. 19 ± 3% and 17 ± 9% inhibition, respectively, in WT mice ( *n* = 7, *N* = 6; [44]) (Fig. 3B). Also the relative small inhibitory effect of MK on the depolarization produced by the just subthreshold KCl stimuli (Fig. 3C) was similar in FHM1 and WT mice (17 ± 13% and 16 ± 6% inhibition of the 1st and 2nd peak amplitudes relative to the resting potential, respectively, in FHM1 mice (*n* = 6, *N* = 6) vs. 15 ± 1% and 15 ± 4% inhibition, respectively, in WT mice (*n* = 3, *N* = 3; [44]) (t-test: *p* = 0.7 and 0.7, respectively). As in WT mice, MK eliminated the later shoulder of the just subthreshold depolarization (Fig. 3C) and shortened its duration by a similar extent in FHM1 and WT mice (27 ± 14% in FHM1 vs. 37 ± 9% in WT [44]; t test: *p* = 0.28)

To display the NMDAR-dependent component (NMDAR-c) of the threshold and just subthreshold depolarizations we subtracted the voltage traces recorded after application of MK to the control voltage traces (Fig. 4A). As found in WT mice [44], the difference traces at CSD threshold show a relatively small NMDAR-dependent component of the early depolarization, followed by a larger late NMDAR-dependent component, which develops after a delay from the application of the KCl stimulus (Fig. 4A). This delay is similar to the delay of CSD initiation: 4.0 ± 1.0 s delay late NMDAR-c; 4.3 ± 0.9 s delay CSD initiation (derived from the beginning of the steep IOS rise in the same experiments) (*n* = 6, *N* = 6) (Fig. 4B).

The difference voltage traces (control - MK) at just subthreshold stimulation show a late NMDAR-dependent component which develops with a similar delay as that at CSD threshold (3.8 ± 1.0 s vs. 4.0 ± 1.0 s, *n* = 6; *N* = 6, paired t test: *p* = 0.68) but has a 78 ± 4% smaller amplitude (*n* = 6, *N* = 6) (Figs. 4A). In contrast with the prolonged duration of the delayed NMDAR-dependent component at CSD threshold, the late NMDAR-dependent component of the depolarization elicited by just subthreshold stimulation decays to zero relatively rapidly (Fig. 4A). The timing of development of this delayed NMDAR-dependent depolarization suggests that, most likely, it underlies the shoulder (and the prolongation of the depolarization) in the control voltage traces recorded in response to subthreshold KCl stimuli near CSD threshold (Figs. 1A and 3C).

Very interestingly, despite the lower stimulation intensities in FHM1 mice (Fig. 2), the delayed NMDAR-dependent components of the neuronal depolarization at just-subthreshold and threshold stimulations have both similar amplitudes in FHM1 and WT mice: 12 ± 6 mV and 54 ± 4 mV at just-subthreshold and threshold stimulations in FHM1 (*n* = 6, *N* = 6) vs. 16 ± 5 mV and 57 ± 4 mV in WT (*n* = 3, *N* = 3; [44]) (Fig. 5A). Moreover, in agreement with the shorter delay for CSD initiation in FHM1 compared to WT mice (Fig. 2), the delay of the late NMDAR-dependent component of the neuronal depolarization relative to the rapid depolarization produced by the threshold KCl stimulus is shorter in FHM1 compared to WT mice, despite the lower stimulation intensity: 4.0 ± 1.0 s in FHM1 (*n* = 6, *N* = 6) vs. 5.4 ± 1.1 in WT (*n* = 7, *N* = 6; [44]) (Fig. 5B).

Overall the data are consistent with the conclusions that (i) in FHM1 mice, as in WT mice, the mechanism underlying the ignition of CSD by a threshold stimulus and not by a just subthreshold stimulus is the delayed opening of a sufficient number of NMDARs (and/or of channels whose opening depends on activation of NMDARs); the time necessary to reach this threshold level of NMDAR activation underlies the slow development of the CSD depolarization and its delay relative to the rapid depolarization produced by the threshold KCl stimulus; (ii) a quantitatively similar threshold level of NMDAR activation is required for CSD initiation at threshold stimulation in FHM1 and WT mice, but this critical level is reached with a stimulus of lower intensity and more rapidly in FHM1 compared to WT mice, thus accounting for the facilitation of CSD initiation in the migraine mouse model.

As reported in WT mice [44], blocking AMPA/kainate receptors in FHM1 mice did not affect the CSD threshold and velocity. The CSD threshold measured after perfusion with NBQX was similar to that in control (130 ± 37 ms vs. 125 ± 10 ms; *n* = 4, *N* = 2). The velocity of CSD propagation was slightly smaller after perfusion with NBQX (3.43 ± 0.27 mm/min vs. 3.98 ± 0.20 mm/min; *n* = 4; *N* = 2, paired t-test *p* = 0.004), but this small decrease is similar to that measured after sham perfusion (14 ± 4% after NBQX vs. 15 ± 10% sham).

The block of CSD initiation by MK, when elicited with CSD threshold KCl stimuli (Fig. 3), leaves open the possibility that largely suprathreshold depolarizing stimuli might be able to ignite CSD even with blocked NMDARs. To establish whether NMDARs are necessary for CSD initiation in FHM1 mice, even when elicited by largely suprathreshold stimuli, we increased the KCl stimulus in the presence of MK up to 3 s duration (as in [44]). While KCl puffs of 0.5 s duration (3.5 times the control CSD threshold) did not elicit CSDs in the presence of MK (*n* = 13, *N* = 11), KCl puffs of 1 s duration (7 times the control CSD threshold) did elicit CSDs in 7 out of 13 experiments (Fig. 6A). However, IOS imaging at different distances from the KCl puffer showed that these CSDs did not propagate throughout the slice, except in one experiment, and in 5 experiments they propagated at a short distance (< 500 μm) from the site of initiation (aborted CSDs, a CSDs). The IOS traces of a representative experiment in which an aCSD was measured after MK-801 perfusion are shown in the left panel of Fig. 6B (lower row traces). In this experiment, after blocking the NMDARs, the steep large IOS change typical of CSD propagated only up to 400 μm (light blue trace) with a clearly much slower rate than in control (see below). When the duration of the KCl stimulus was increased to 3 s (21 times the control CSD threshold), CSDs were elicited in all 13 experiments in the presence of MK (Fig. 6A), although in only 5 of them the CSD propagated throughout the slice; in most of the other experiments (*n* = 7) the CSDs propagated beyond 500 μm (but less than 1 mm, on average up to 630 ± 95 μm; mini CSDs, mCSDs). The IOS traces of a representative experiment in which a mCSD was measured after MK-801 perfusion are shown in the right panel of Fig. 6B (upper row traces). In this experiment, after blocking the NMDARs, the steep large IOS change typical of CSD propagated only up to 800 μm (brown trace) with a clearly much slower rate than in control (see below). The lower row traces in the right panel of Fig. 6B are from a representative experiment in which, after MK-801 perfusion, a CSD propagating throughout the slice (but with a much lower velocity than in control: see below) was measured. In contrast, in WT mice, the same KCl stimulus (3 s duration) did not elicit CSDs in the presence of MK, despite the fact that it depolarized the neuronal membrane even more than the control CSD [44]. Interestingly, as found in WT mice [44], the block of the Ca_V_ channels with Ni^2+^ (5 mM) prevented CSD initiation by largely suprathreshold (3 s) stimuli in FHM1 mice (*n* = 6, *N* = 3) (Fig. 6A). Thus, in contrast with WT mice, in FHM1 mice with gain-of-function of Ca_V_2.1 channels, other (Ca_V_2.1-dependent) processes, besides activation of NMDARs, may contribute to CSD initiation by intense stimulation.

The CSDs elicited in FHM1 mice by largely suprathreshold (relative to control) stimuli after blocking NMDARs propagated at a much slower rate than the control CSDs: 1.42 ± 0.3 mm/min in MK vs. 3.67 ± 0.6 mm/min in control (*n* = 11, *N* = 9, considering together CSDs and mCSDs, since they propagated at the same rate: mCSDs 1.41 ± 0.3 mm/min, *n* = 7, *N* = 5; CSDs 1.42 ± 0.3 mm/min, *n* = 4; *N* = 4; t test, *p* = 0.98) (Figs. 6B and 7A).

In WT mice, despite the block of CSD initiation by MK, the largely suprathreshold (1 s and 3s duration) stimuli depolarized the neurons close to the KCl puffer in the presence of MK even more than the control CSD [44]. Therefore, to study the properties of the CSD depolarizations elicited in FHM1 mice after blocking NMDARs, we recorded the voltage changes of neurons located at ≥ 600 μm from the KCl puffer in response to CSD threshold KCl puffs in control and to suprathreshold KCl stimuli after MK perfusion. As shown by the overlapped voltage traces in Fig. 7B and C, the CSDs elicited after inhibition of NMDARs are quite different from the control CSDs. Both the peak voltage and the duration of the CSD depolarizations in the presence of MK are lower than those of the control CSD (peak voltage: -18 ± 6 mV vs. − 1.6 ± 2.1 mV, *n* = 4, *N* = 3; HW: 60 ± 11 s vs. 87 ± 18 s, *n* = 4, *N* = 3) (Fig. 7B). The expanded CSD traces in Fig. 7C show that also the kinetics of the voltage changes are different. Both in control and in the presence of MK a prodromal slow neuronal depolarization precedes by few seconds a much faster depolarization, and two different phases can be distinguished in the fast depolarization. However, the prodromal slow depolarization develops more slowly in the presence of MK and also both phases of the fast depolarization are slower after NMDAR block (Fig. 7C).

The voltage and IOS changes measured simultaneously at the location of the recorded neuron (plotted on the same time scale in Fig. 8A to facilitate comparison) show that, in both control and after NMDAR block, the prodromal neuronal depolarization begins several seconds ahead of CSD initiation (as measured from the beginning of the steep IOS rise). After block of NMDARs, the delay with which CSD initiates relative to the beginning of the prodromal neuronal depolarization is more than two times longer than in control (15 ± 1 s vs. 6.8 ± 2.0 s, *n* = 4, *N* = 3) (Fig. 8B, upper left panel). This longer delay appears to account for the difference in delay of CSD initiation relative to the time of the high KCl puff application in control and after NMDAR block (24 ± 3 s in the presence of MK vs. 14 ± 2 s in control, *n* = 4, *N* = 3) (Fig. 8B, upper right panel). Indeed, the delay of the beginning of the prodromal depolarization relative to the KCl puff was not significantly different in control and after NMDAR block (7.0 ± 2.9 s and 9.4 ± 3.2 s, respectively, *n* = 4, *N* = 3) (Fig. 8B, lower left panel). The longer delay with which CSD initiates relative to the beginning of the prodromal depolarization appears to substantially contribute to the slower velocity of CSD propagation after NMDAR block. Indeed, there is a good inverse linear correlation between the velocity of CSD propagation and the delay with which CSD initiates relative to the beginning of the prodromal neuronal depolarization in individual experiments (both in control and in the presence of MK) (Fig. 8C).

An interesting observation stemming from the comparison of the voItage and IOS changes measured simultaneously at the location of the recorded neuron located far from the KCl puffer (Fig. 8A) is that the beginning of CSD (as measured from the beginning of the steep IOS rise) coincides with the beginning of the second phase of the steep depolarization produced by either threshold stimulation in control or suprathreshold stimulation after NMDAR block. Interestingly, the neuronal membrane potential at which CSD and the second phase of the fast depolarization begin has the same value in control and after NMDAR block (− 45 ± 4 mV in control vs. − 45 ± 5 mV in the presence of MK, *n* = 4, *N* = 3) (Fig. 8B, lower right panel). The fast regenerative CSD depolarization above this value is slower in the presence of MK (7.3 ± 3.0 mV/s in MK vs. 22 ± 2 mV/s in control, *n* = 4, *N* = 3) (Fig. 8D, left panel), suggesting that this contributes to the slower velocity of CSD propagation after NMDAR block (in addition to the longer time needed to reach the voltage threshold for CSD initiation after the beginning of the prodromal neuronal depolarization). Accordingly, the propagating fast IOS change typical of CSD, measured simultaneously at the location of the recorded neuron, is slower after NMDAR block (6.1 ± 3.3 vs. 21 ± 4 IOS/s, *n* = 7, *N* = 5) (Fig. 8D, center panel). Considering the values of individual experiments in both control and after MK, there is a good linear correlation between the velocity of CSD propagation and the kinetics of both the propagating IOS change (IOS/s) and the second phase of the CSD depolarization (mV/s) (Fig. 8D, right panel). The same linear function best fitted the IOS (continuous line with slope 0.13 ± 0.01 in Fig. 8D) and the second phase of the CSD depolarization (dashed line with slope 0.13 ± 0.02 in Fig. 8D).

## Discussion

Our investigation of the mechanisms underlying CSD initiation by focal application of high KCl in acute cerebral cortex slices provides evidence that in FHM1 mice, as in WT mice, the mechanism underlying the ignition of CSD by a threshold stimulus and not by a just subthreshold stimulus is the delayed opening of a sufficient number of NMDARs (and/or of channels whose opening depends on activation of NMDARs). The time necessary to reach this threshold level of delayed NMDAR activation underlies the slow development of the CSD depolarization and its delay relative to the rapid depolarization produced by the KCl stimulus. This threshold level of delayed NMDAR activation (which is critical for making the depolarization regenerative and initiate CSD at threshold stimulation) is similar in FHM1 and WT mice, despite the fact that the threshold stimulation for CSD initiation is almost 50% lower in FHM1 mice. These findings support the idea that a threshold level of NMDARs activation is necessary for CSD ignition regardless of genotype. However, in FHM1 mice, this critical level is reached with depolarizing stimuli that are largely subthreshold for NMDAR activation and CSD initiation in WT mice. Moreover, despite the lower stimulation intensities in FHM1 compared to WT mice, the time necessary to reach the threshold level of delayed NMDAR activation and the delay of CSD initiation are both shorter in FHM1 mice. Our findings are consistent with the previous evidence that a threshold level of glutamate (and glutamate “plumes”) is necessary for CSD ignition regardless of genotype [31], and support the idea that the critical mechanism downstream of the glutamate elevation is the activation of NMDARs.

An interesting question concerns the mechanisms underlying the long delay of CSD initiation relative to the rapid depolarization produced by the KCl stimulus. This long delay reflects the long time required to reach the threshold level of NMDARs activation necessary for CSD initiation. Overall the findings are consistent with the hypothesis that these delays reflect the time required to reach the threshold level of glutamate increase necessary for CSD initiation. Indeed, glutamate imaging at the site of CSD initiation in awake WT head-fixed mice revealed a slow rise of extracellular glutamate and an increase in the frequency of glutamate “plumes”, which began at a time before CSD onset by threshold stimulation (about 6 s) [31] quite similar to the delay required to reach the threshold level of NMDAR activation in our WT experiments [44]. When, after this time, both the glutamate rise and the “plumes” frequency reached a critical threshold level, CSD ignited [31]. Since the glutamate “plumes” in layer 1 can be considered as a marker of inefficient glutamate clearance [31], one can hypothesize that the delay with which the threshold levels of glutamate and NMDAR activation are reached may reflect the time necessary to reach a critical level of impairment of glutamate clearance, due to decreased cycling rate of glutamate transporters consequent to the depolarization, the extracellular K^+^ increase and the intracellular accumulation of Na^+^ and glutamate produced by the KCl stimulus [44]. The finding that the delays for attainment of the critical threshold level of NMDAR activation and for CSD initiation are both shorter in FHM1 mice (which show enhanced glutamate release at cortical synapses [35, 46]), appears consistent with this hypothesis. Also consistent with the hypothesis is the previous finding that the rise of extracellular glutamate to the threshold level necessary for CSD initiation is faster in FHM2 mice (which show reduced rate of glutamate clearance at cortical synapses [31, 36]).

It remains possible that other Ca_V_2.1 channel-dependent mechanisms may contribute to the delay of CSD initiation relative to the rapid depolarization produced by the KCl stimulus and to its shortening in FHM1 mice carrying a gain-of-function mutation in the Ca_V_2.1 channel. We have previously shown that, in WT mice: (i) the opening of Ca_V_ channels contributes to the first peak and is (indirectly) responsible for the second peak of the early threshold depolarization preceding CSD; (ii) CSD initiation at threshold depolarization is prevented by inhibition of the Ca_V_ channels (with Ni^2+^) and also by specific inhibition of the Ca_V_2.1 channels (with ω-AgaIVA) [44]. In FHM1 mice, despite the smaller KCl stimuli, the amplitudes of the second peak of the early threshold depolarization preceding CSD and of the second peak of the subthreshold depolarization are quantitatively similar to those in WT mice, and also similar to WT is their relatively small (< 20%) inhibition by MK-801. Thus, similar neuronal depolarizations in FHM1 and WT mice are produced by depolarizing KCl stimuli of much lower intensity in FHM1 compared to WT mice. This indicates that, for a given KCl stimulus, in FHM1 mice, the gain of function of the Ca_V_2.1 channel leads, not only to the increased glutamate release underlying the relatively larger activation of NMDARs, but also to a larger activation of the cationic channels underlying most of the second peak of the subthreshold and early threshold depolarizations preceding CSD. The opening of these cationic channels depends on and temporally follows that of Ca_V_ channels, whose location (presynaptic or postsynaptic) remains unclear [44]. The nature of these Ca_V_2.1-dependent cationic channels and the role they may possibly play in CSD initiation remain also unclear. However, their larger activation in FHM1 mice might contribute, to some extent, to the shorter delay of CSD initiation in the migraine mutants (and perhaps also to the lower CSD threshold).

Overall the findings in FHM1 mice (as well as in FHM2 and MA mice [15]) suggest a model of CSD initiation (in normally metabolizing brain tissue) in which a threshold depolarizing stimulus leads to enough Ca_V_-dependent glutamate release to overwhelm the reuptake capacity of the astrocytic glutamate transporters, thus leading to cooperative activation of (synaptic and extrasynaptic [37]) NMDARs above the critical level necessary to generate a net self-sustaining inward current. This makes the neuronal depolarization and the K^+^/glutamate elevations self-regenerative and initiates the positive feedback cycle that ignites CSD [4, 43]. There is a reciprocal relationship between K^+^ and glutamate that is likely essential to CSD initiation [15]: glutamate binding to post-synaptic receptors results in K^+^ release (particularly through NMDARs: [48, 49]), and extracellular K^+^ further depolarizes the postsynaptic neuronal membrane as well as the presynaptic terminals responsible for glutamate release [49], and decreases the efficiency of glutamate reuptake by both depolarizing the astrocyte membranes and reducing the K^+^ electrochemical gradient [50–52]. This amplificatory cycle of glutamate/K^+^ release is potentiated in the genetic mouse models of migraine as a consequence of either increased glutamate release (in FHM1 and MA mice [35, 38, 46]) or reduced rate of glutamate clearance (in FHM2 mice [31, 36]), resulting in increased susceptibility to CSD because the CSD- threshold levels of glutamate and NMDAR activation are reached with stimuli of lower intensity and more rapidly.

Shedding light on the mechanisms underlying the facilitation of CSD initiation in a genetic mouse model of migraine, our findings give insights into potential mechanisms of CSD initiation in the brain of migraineurs. Considering the evidence that CSD is most easily inducible in the upper dendritic layers of neocortex [4, 12, 53], our findings suggest that ignition of a “spontaneous” CSD in the brain of FHM and MA patients is favoured by conditions leading to excessive activation of synaptic and extrasynaptic NMDARs in the apical dendrites of cortical pyramidal cells. This would probably require hyperactive excitatory synapses in upper layers (high-frequency repetitive or synchronous activity of a sufficient number of excitatory synapses in which glutamatergic transmission is genetically potentiated) as well as disinhibition of apical dendrites [46, 54, 55]. We can hypothesize that this may occur, in certain conditions, as a consequence of dysfunctional regulation of the E/I balance in specific neural circuits in the migraine brain [56]. Consistent with this hypothesis, there is evidence of dysfunctional regulation of the E/I balance in cortical microcircuits in FHM1 and MA mice [38, 46, 57, 58]. However, the relevant circuits and conditions in which dysfunctional regulation of the E/I balance may favour initiation of spontaneous CSDs remain unclear. In this respect, it seems important to consider the episodic nature of CSD, and to note that its episodic onset in the migrane brain likely depends on the presence of one or more triggering factors, on brain neuromodulatory state and perhaps some metabolic compromise [15]. As a matter of fact, spontaneous CSDs were observed very rarely and only in a small subset of FHM1 mice with implanted electrodes in the cortex [34].

An unexpected, interesting finding of our study is that, in contrast with WT mice [44], CSD can be initiated by very intense, largely suprathreshold stimulation after block of NMDARs in FHM1 mice, although it cannot be initiated by this intense stimulation after block of the Ca_V_ channels (as in WT mice [44]). Thus while, no matter the intensity of stimulation, Ca_V_ channels appear necessary for initiation of CSD (by focal high KCl stimuli), the necessity of NMDARs for CSD initiation can be overcome by largely suprathreshold stimuli in FHM1 mice. The NMDAR-independent but Ca_V_2.1-dependent channels/processes involved in CSD initiation by very high intensity stimulation in FHM1 mice remain unknown.

The slower velocity of propagation of the CSDs elicited in FHM1 mice by very high intensity stimulation in the presence of MK-801 and the short distance at which most of them propagate point to the important role of NMDARs in CSD propagation besides CSD initiation. Our simultaneous recordings of the membrane potential of neurons located far from the site of CSD initiation and of the IOS change at the site of the recorded neurons provide insights into the mechanisms of CSD propagation and the role of NMDARs in these mechanisms. The slow rate of CSD propagation implies that it is mediated by diffusion of a chemical substance [4, 12]. While there is a large increase of the extracellular concentration of both K^+^ and glutamate during the CSD depolarization, most evidence points to K^+^ rather than glutamate as the diffusing substance that initiates CSD into contiguous gray matter, thus leading to its propagation [4, 59]. We have found that both in control and in the presence of MK-801, the neurons located far from the KCl puffer begin to slowly depolarize several seconds before the beginning of the propagating CSD (as measured from the beginning of the steep IOS rise at the location of the recorded neuron). This finding is consistent with in vivo evidence that in the cerebral cortex a [K^+^]e rise precedes by several seconds the beginning of the propagating CSD (as measured from the beginning of the negative DC potential change) [59, 60], and is consistent with the interpretation that this [K^+^]e rise initiates the prodromal neuronal depolarization. Interestingly, the prodromal neuronal depolarization that precedes the CSD depolarization is much slower in the presence of MK-801 compared to control, and the delay with which CSD initiates relative to the beginning of the prodromal depolarization is more than two times longer in the presence of MK-801 compared to control. However, the neuronal membrane potential at which CSD initiates is similar in control and after NMDAR block. This is consistent with the interpretation that, in FHM1 mice, the delay of the CSD depolarization relative to the beginning of the prodromal depolarization reflects the time necessary to open cationic channels, including NMDARs, in a number sufficient to generate a net self-sustaining inward current, which makes the neuronal depolarization and the [K^+^]e increase (and consequent glutamate release) self-regenerative, and thus initiate the positive feedback cycle that ignites CSD far from the original initiation site (if the removal of K^+^ and glutamate do not keep pace with release) [4, 43]. The finding that, after initiation of the positive feedback cycle, the fast regenerative CSD depolarization is slower in the presence of MK-801 indicates that NMDARs play an important role also in sustaining the CSD positive feedback cycle (besides in initiating it). This important double role of NMDARs explains the slower velocity of propagation of the CSDs elicited with blocked NMDARs in FHM1 mice (and, most likely, also the short distance at which most of them propagate).

Several previous pharmacological studies have shown that the block of the NMDARs in WT animals completely inhibits CSD measured far from the focal (threshold and suprathreshold) depolarizing stimuli ( [4] and references therein). Although most of them, cannot distinguish whether NMDARs are necessary for CSD initiation or CSD propagation (or both), there is some direct evidence that, in WT animals, the block of NMDARs prevents CSD propagation [61]. Our finding that this does not occur in FHM1 mice (when very intense, largely suprathreshold, stimulation overcomes the necessity of NMDARs for initiation of CSD at the site of focal stimulation) suggests that, besides NMDARs activation, other Ca_V_2.1-dependent channels/processes are involved in initiation and maintenance of the positive feedback cycle that mediates CSD propagation. The nature of these channels/processes and whether they are the same as those involved in CSD initiation with very intense stimulation in FHM1 mice remain unknown.

## Conclusions

We have shown that a delayed activation of NMDARs above a critical threshold level is necessary for CSD initiation by brief, focal high-KCl stimuli in FHM1 mice. This threshold level of NMDAR activation is quantitatively similar in FHM1 and WT mice, but it is reached with a stimulus of much lower intensity and more rapidly in FHM1 mice, thus accounting for the facilitation of CSD initiation in this migraine mouse model. Our findings are consistent with the previous evidence for a threshold level of glutamate (and glutamate “plumes”) necessary for CSD ignition regardless of genotype, and support the idea that the critical mechanism downstream of glutamate elevation is the activation of NMDARs. While Ca_V_ channels appear necessary for initiation of CSD (no matter the intensity of stimulation), the necessity of NMDARs for CSD initiation can be overcome by largely suprathreshold stimuli in FHM1 mice; however, these NMDAR-independent CSDs propagate much more slowly than the control CSDs, due to both a longer time needed to reach the threshold for CSD initiation after the beginning of the prodromal neuronal depolarization and a slower regenerative CSD depolarization. Our findings give insights into potential mechanisms of initiation of “spontaneous” CSDs in the brain and into the unknown mechanisms which make the brain of migraineurs susceptible to CSD.

## Data Availability

Most of the data generated or analysed during this study are included in this published article and all data are available from the corresponding author on reasonable request.

## Abbreviations

CSD: cortical spreading depression
FHM1: familial hemiplegic migraine type 1
NMDARs: glutamate NMDA receptors
FHM: familial hemiplegic migraine
FHM2: familial hemiplegic migraine type 2
α_2_NKA: α_2_ Na^+^, K^+^ ATPase
MA: migraine with aura
FASPS: familial advanced sleep syndrome
WT: wild-type
Ca_V_ channels: voltage-gated Ca^2+^ channels
IOS: intrinsic optic signal
L2/3: layer 2/3
HW duration: duration at half amplitude
MK: MK-801
NMDAR-c: NMDAR-dependent component
